# Cerebral metabolism in major depressive disorder: a voxel-based meta-analysis of positron emission tomography studies

**DOI:** 10.1186/s12888-014-0321-9

**Published:** 2014-11-19

**Authors:** Liang Su, Yiyun Cai, Yifeng Xu, Anirban Dutt, Shenxun Shi, Elvira Bramon

**Affiliations:** Department of Psychiatry, Huashan Hospital, Fudan University, School of Medicine, No. 12 Wulumuqi Road (middle), Shanghai, 200040 People’s Republic of China; Department of Psychosis studies, Institute of Psychiatry, King’s College London, De Crespigny Park, London, SE5 8AF UK; Shanghai Mental Health Center, Shanghai Jiao Tong University School of Medicine, Shanghai, China; Mental Health Sciences Unit & Institute of Cognitive Neuroscience, University College London, London, UK

**Keywords:** Major depressive disorder, Positron emission tomography, Meta-analysis, Activation likelihood estimation

## Abstract

**Background:**

Major depressive disorder (MDD) is a common mental illness with high lifetime prevalence close to 20%. Positron emission tomography (PET) studies have reported decreased prefrontal, insular and limbic cerebral glucose metabolism in depressed patients compared with healthy controls. However, the literature has not always been consistent. To evaluate current evidence from PET studies, we conducted a voxel-based meta-analysis of cerebral metabolism in MDD.

**Method:**

Data were collected from databases including PubMed and Web of Science, with the last report up to April 2013. Voxel-based meta-analyses were performed using the revised activation likelihood estimation (ALE) software.

**Results:**

Ten whole-brain-based FDG-PET studies in MDD were included in the meta-analysis, comprising 188 MDD patients and 169 healthy controls. ALE analyses showed the brain metabolism in bilateral insula, left lentiform nucleus putamen and extra-nuclear, right caudate and cingulate gyrus were significantly decreased. However, the brain activity in right thalamus pulvinar and declive of posterior lobe, left culmen of vermis in anterior lobe were significantly increased in MDD patients.

**Conclusion:**

Our meta-analysis demonstrates the specific brain regions where possible dysfunctions are more consistently reported in MDD patients. Altered metabolism in insula, limbic system, basal ganglia, thalamus, and cerebellum and thus these regions are likely to play a key role in the pathophysiology of depression.

## Background

The application of neuroimaging techniques, such as Positron Emission Tomography (PET), to the study of major depressive disorder can contribute to the development of pathophysiologic models and to the understanding of the neurobiology of the disease [1]. Both fMRI and PET enable creation of functional connectivity maps of distinct spatial distributions of brain regions. However, fMRI and PET measure changes in the composition of blood and brain metabolism, respectively. Only PET allows the measurement of cerebral glucose metabolism in vivo [2].

Some PET studies typically showed that depressed subjects have reduced lateral prefrontal metabolism and increased medial prefrontal and subgenual cingulate metabolism [3,4]. Also some studies reported lower regional glucose metabolism in mood disorders in dorsolateral, ventral subgenual and doromedial prefrontal cortical regions. Our team found that, compared with healthy controls, depression patients demonstrated decreased regional cerebral glucose metabolism rate in bilateral caudate, superior temporal gyrus, inferior frontal gyrus, middle frontal gyrus, right lingual gyrus and left cingulate gyrus, superior temporal gyrus [4]. However, these studies had inconsistent results, the other studies also found that MDD patients showed greater glucose uptake in ventrolateral prefrontal cortical and paralimbic regions compared to healthy subjects [5-7].

In a previous meta-analysis of neuroimaging studies using PET, Single Photon Emission Computed Tomography (SPECT) and functional Magnetic Resonance Imaging (fMRI) in major depression, Fitzgerald et al [8] reported that the dorsal cingulate gyrus, middle and dorsolateral prefrontal cortex, insula and superior temporal gyrus are hypoactive at rest and show a lack of activation during negative mood states that improves with SSRI treatment. According to Delaveau et al [9] antidepressants increase the activation of dorsolateral, dorsomedial and ventrolateral prefrontal cortices in one PET study and eight fMRI studies; whereas another meta-analysis of six MRI and four PET studies by Sacher et al [10] claims that the activation of the amygdala, hippocampus, parahippocampal region, ventral anterior cingulate cortex, orbitofrontal cortex, and insula is decreased. These meta-analyses have dealt with literature from a variety of functional neuroimaging technologies and this may have contributed to the apparent inconsistent findings.

Meta-analyses are essential for summarizing the literature, comparing results in a standardized fashion, and quantifying the relationship between study characteristics and findings [10]. However, it is difficult to compare and combine data across studies using different imaging methods such as PET and fMRI [11]. We perform the new ALE meta-analysis which widened of samples by including Chinese studies. Thus the present meta-analysis will focus only on a more homogeneous literature of studies comparing cerebral glucose metabolism measured with PET in patients with major depressive disorder and controls.

## Methods

### Literature search and inclusion criteria

According to PRISMA (Preferred Reporting Items for Systematic reviews and Meta-Analyses) guidelines [12], search strategy and selection criteria Data were identified by electronic searches of PubMed (January 1978 to April 2013), the Chinese ‘MEDLINE’ China Biological Medicine Database [13] (CBM-disc 1979 to April 2013), China National Knowledge Infrastructure (CNKI 1996 to April 2013), Cochrane Library and Web of Science (1978 to April 2013), with the terms “Depressive”, “Depression”, “PET”, “positron emission tomography”, “FDG” and “18 F-Fluorodeoxyglucose”. References related to the identified articles were hand searched as reported in our previous study [14]. We only included studies comparing major depression patients and control subjects.

All studies had to meet the following criteria: (1) peer-reviewed original research articles, (2) MDD diagnosis according to internationally recognized diagnostic criteria, (3) reported age matched control group, (4) measured glucose metabolism (PET), and (5) results reported as coordinates in a normalized standard stereotactic space [Talairach or Montreal Neurological Institute (MNI) reference system], (6), at least five subjects in each of the patient and healthy comparison groups [15]. Studies had to be available in English or Chinese, and involve adult participants.

Studies reporting only results pre- and post-antidepressive treatment without healthy control group, or reporting post-medication effects only were excluded. Studies using region of interest (ROI) method were excluded [5] because they did not report brain coordinate and we only included studies using a voxel-by-voxel image analysis method. The literature search, selection of studies according to the inclusion and exclusion criteria, and compilation of coordinates for the contrasts were performed independently by two investigators.

### Meta-analysis methods

Meta-analyses were performed using the revised activation likelihood estimation (ALE) software implemented in GingerALE 2.0 http://www.brainmap.org/ale/ [16-20]. An improved version of this method was published by Eickhoff et al [16]. The input coordinates were weighted to form estimates of activation likelihood for each intracerebral voxel. The activation likelihood of each voxel in standard space was then combined to form a statistic map of the ALE score at each voxel. We calculated ALE maps separately for FDG-PET studies.

Voxel-by-voxel analyses may increase the likelihood of reporting false positive results, since they require tens or hundreds of thousands of statistical comparisons. This was addressed by correcting P values for the number of independent resolution elements within a search area and by constraining the size of the search area [21]. The resulting ALE map was threshold at p <0.05 (corrected for multiple comparisons by false discovery rate (FDR), and a minimum cluster size of supra threshold voxels exceeding 100 mm^3^ was imposed. ALE results were overlaid onto an optimized individual anatomical T1-template http://www.brainmap.org/ale/Colin27_T1_seg_MNI_2x2x2.nii.gz [22].

### Primary literature

**Figure 1:**
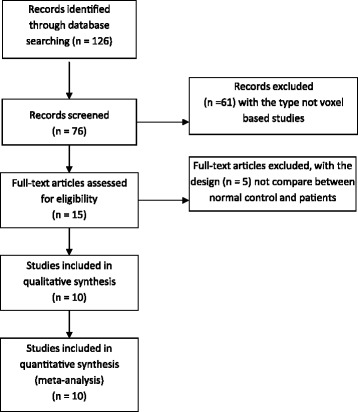
**Flow Diagram of voxel-based meta-analysis of PET studies in MDD.**

There were 126 studies initially identified. After limiting the results by the criteria described above, 10 whole-brain-based FDG-PET studies in MDD were considered eligible to enter the meta-analysis. These included a total of 188 patients and 169 healthy controls as showed in Figure 1. Compared MDD patients and control samples, all of the 10 PET studies reported decreased brain activity, but only 8 studies reported increased metabolism changes in patients. The average sample size per study was 18.8 ± 9.4 patients and 16.9 ± 8.92 controls. The gender ratio of the patient samples was approximately even (54.1% females). More detailed information on included studies can be found in Table 1.

**Table 1:** **Details of the 10 primary studies included in this meta-analysis**

**Year**	**Author**	**Disorder**	**Patients**			**Controls**			**Depression**	**PET Software And**	**Medication**
			Sample Size	Gender	Mean Age (SD)	Sample Size	Gender	Mean Age (SD)	Severity HDRS)	FWHM	
2001	Saxena [23]	MDD	27	13 M, 14 F	38.2(11.1)	17	8 M, 9 F	32.5(11.7)	17 20.8(5.0)	SPM 96, 16 mm	*
2001	Brody [24]	MDD	24	11 M, 13 F	38.9(11.4)	16	8 M, 8 F	35.6(18.3)	17, 19.4(5.5)	SPM 96, 10 mm	**
2001	Kennedy [25]	MDD	13	13 M	36(10)	24	24 M	31.7(6.7)	17 22.4(3.6)	SPM 96, 8 mm	treated with antidepressants
2002	Kimbrell [7]	MDD	37	13 M, 24 F	43.4(13.0)	37	13 M, 24 F	43.4(12.5)	28, 16.9(8.3)	SPM 95, 10 mm	medication-free
2003	Kegeles [26]	MDD	19	6 M, 13 F	16(11)	10	4 M, 6 F	39(19)	17, 22.3(3.9)	SPM 96, 8 mm	medication-free at least 14 days
2005	Zhang [27]	MDD	14	4 M, 10 F	43.8(9.3)	11	3 M, 8 F	43.0(7.8)	17, 27.8(6.2)	SPM 2b, 8 mm	treated with antidepressants
2006	Su [4]	MDD	6	2 M, 4 F	71(6)	10	4 M, 6 F	67.6(2.9)	24, 39.7(3.2)	SPM 2, 10 mm	first episode and medication-free
2007	Aihara [28]	MDD	24	9 M, 15 F	52.4(13.4)	23	8 M, 15 F	54.8(12.6)	21, 32.0 ± 6.8	SPM 99, 10 mm	medication-free
2009	Smith [29]	MDD	16	6 M, 10 F	65.3(9.1)	13	5 M, 8 F	67.4(7.4)	24, 26.0(3.5)	SPM 99, 8 mm	medication-free
2009	Wu [30]	MDD	8	5 M, 3 F	47.1(9.3)	8	5 M, 3 F	45.1(8.2)	17, 23.1(3.9)	SPM 2b, 8 mm	medication-free

Note: FWHM = Full Width at Half Maximum, SPM = Statistical Parametric Mapping, M = man, F = female, HDRS = Hamilton Depression Rating Scale.

*Either treatment naïve or off medication for more than 1 year.

**Either treatment naïve or off medication at least 2 weeks.

## Results

### Summarize the studies included comparison of MDD and normal controls

Summarize the studies included along with the total number of activation foci observed, ALE analyses were separately carried out ten increased 10 studies (54 foci) and eight decreased studies (41 foci) brain metabolism in MDD patients.

### Decreased metabolism in MDD

**Figure 2:**
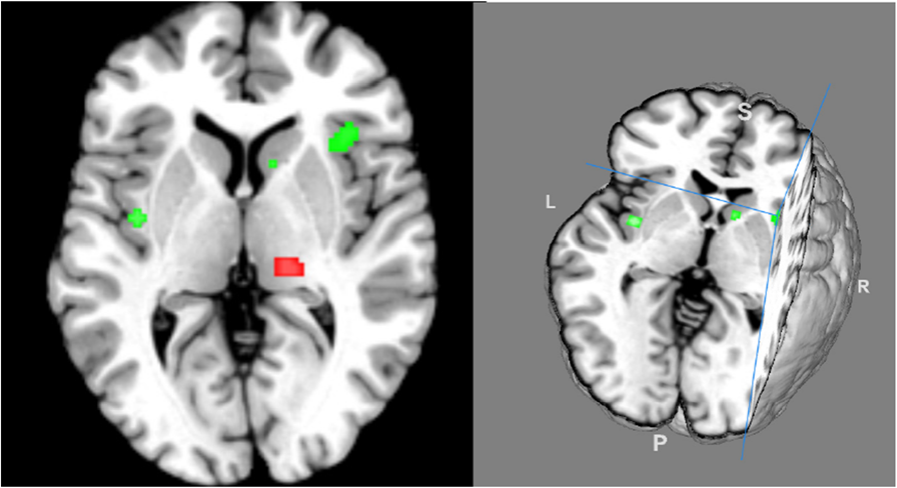
**3D (right) and 2D (left) ALE map representing the decreased metabolism (color in green) of insula, limbic system and basal ganglia, and the increased metabolism of thalamus, and cerebellum (color in red) in MDD patients compared to normal controls (P<0.05, FDR-corrected) (produced by Mango Image Viewer,** **http://www.brainmap.org/tools.html** **).**

As illustrated in Figure 2 and Table 2, we found significant decreases in cluster size within the cortico-limbic circuit, a network relevant to MDD, including both insula (BA 13, right and insula, cluster size: 536 and 152 mm^3^, with a maximum ALE value of 0.0111 and 0.0103, respectively), left extra-nuclear (BA 13, cluster-size; 312 mm^3^, with a maximum ALE value of 0.0113), right caudate head (cluster-size; 176 mm^3^, with a maximum ALE value of 0.009), left lentiform nucleus putamen (cluster-size; 152 mm^3^, with a maximum ALE value of 0.0103) and right cingulate gyrus (BA 31; cluster size: 152 mm^3^, maximum ALE value of 0.0103).

**Table 2:** **Results from ALE-analysis of PET studies in MDD patients (P < 0.05 corrected for multiple comparisons by false discovery rate)**

	**Hemisphere**	**BA**	**volume (m3)**	**x**	**y**	**z**	**Peak ALE value**
**A. Areas of decreased glucose metabolism in MDD patients**							
Insula	R	13	536	36	22	0	0.01111734
Extra-Nuclear	L	13	312	-44	8	-14	0.01131126
Caudate Head	R		176	12	20	-6	0.00999559
Insula	L	13	152	-40	-6	0	0.01027422
Lentiform Nucleus. Putamen	L		152	-32	-16	8	0.01027356
Limbic Lobe. Cingulate Gyrus	R	31	152	12	-26	40	0.01027141
**B. Areas of increased glucose metabolism in MDD patients**							
Thalamus. Pulvinar	R		304	16	-24	4	0.01226161
Cerebellum Anterior Lobe. Culmen of Vermis	L		296	0	-66	-6	0.01194303
Cerebellum Posterior Lobe. Declive	R		264	20	-58	-12	0.01064389

### Increased metabolism in MDD

As illustrated in Figure 2 which was produced by Mango Image Viewer (http://www.brainmap.org/tools.html) we found significant increases in cluster size relevant to MDD, including the right thalamus pulvinar (cluster size: 304 mm^3^, with a maximum ALE value of 0.0123), left culmen of vermis in the anterior lobe cortex (cluster-size: 296 mm^3^, maximum ALE value of 0.0119), and right declive of the posterior lobe (cluster size: 264 mm^3^, with a maximum ALE value of 0.0106). The peak coordinates are shown in the Table 2.

## Discussion

This meta-analysis was undertaken to test current theoretical models of MDD that propose abnormal ventral frontal, basal ganglia and limbic activity on FDG-PET studies. Our main results are that patients with MDD have decreased brain metabolism in bilateral insula, right cingulate gyrus and caudate and left putamen and extra-nuclear. In the meta-analysis we also found increased glucose metabolism in right thalamus and posterior lobe declive, and left culmen of vermis in MDD patients. The results showed that in MDD patients the cluster size of reduced glucose metabolism was higher than the cluster size of increased glucose metabolism. Our results showed that depression patients had a dysfunction regions including hypoactive metabolism in insula and limbic system with compensatory increased activity in thalamus.

The meta-analysis results demonstrated that MDD patients exhibit decreased brain activation on bilateral insula, particularly in the right insula. Using fMRI Wiebking et al [31] found that MDD patients showed reduced neural activity during rest periods, particularly in the bilateral anterior insula. Delavearu et al, in a previous meta-analysis also found decreased insula activation in MDD patients [9]. Using [^15^O-H^2^O] positron emission tomography, Savitz et al. [32] also found that the left posterior insula is a relevant region in MDD. These results, using different neuroimaging methods, consistently show that the insula plays an important role in the neuropathology of depression.

The results also verify that the limbic system including cingulate gyrus and the basal ganglia including caudate head, lentiform nucleus putamen and extra-nuclear had abnormal brain metabolism in MDD patients. Results from a previous study indicated that in the context of untreated symptoms of depression these regions showed decreased activation and that limbic right posterior cingulate and right basal ganglia activation were seen after anti depression treatment [33]. Our meta-analysis showed the limbic lobe and basal ganglia decreases are a similar to those reported by Lai et al. [34] and Tol et al. [35]. The results suggest that the limbic system and basal ganglia might be the targets of treatment, and that antidepressants may exert their benefits by means of increasing brain metabolism in these regions [36].

The second interesting result from our analysis is that certain regions are increased in MDD patients. Some studies showed that MDD patients had higher brain activity in thalamus by FDG-PET [24] and resting-state fMRI [37]. Our results also showed increased metabolism at the culmen of vermis in the cerebellum’s anterior lobe, a region where abnormalities have been previously reported in geriatric depression using MRI and voxel-based morphometry [38]. Again we found increased metabolism in the declive (posterior lobe in cerebellum), which is involved in verbal working memory and has not received much previous attention in depression [39]. We found that the cluster size of reduced glucose metabolism was higher than the cluster size of increased glucose metabolism in MDD patients. One plausible way of integrating these findings is that decreased metabolism in insula, limbic system and basal ganglia is compensated by an increased metabolism in the thalamus and cerebellum [10].

Previously published meta-analyses in neuroimaging studies on depression did not include Chinese patients whereas our study also includes papers published in Chinese therefore enlarging the overall sample considered. Stuhrmann et al [40] analyzed studies of fMRI on emotional face processing in acutely depressed patients compared with healthy controls. The abnormalities in MDD patients showed mood-congruent processing bias particularly in the amygdala, insula, parahippocampal gyrus, fusiform face area, and putamen. Chen et al [15] also used fMRI and found that depressed patients with bipolar disorder had increased brain activity in caudate, precentral gyrus and decreased inferior frontal gyrus. Fitzgerald et al [8] used 3 different methods including fMRI, PET, and SPECT while Sacher et al [10] and Delaveau et al [9] used fMRI and PET.

Although there is a significant overlap between studies already meta-analyzed by Fitzgerald et al. [8] and Sacher et al. [10] and those here meta-analyzed [namely: Brody et al. [24], Kennedy et al. [25], Kimbrell et al. [6] and Saxena et al. [23], for a total of 102 patients and 94 controls (196 subjects)], the authors here added more six studies, encompassing more 87 patients and 75 controls (162 subjects), for an increase of 82%. In addition, although in their methodologies Sacher et al. and Delaveau et al. address separately fMRI and PET studies, but their analyses were carried out without distinguishing enough modalities. Rest fMRI is also an important tool to detect brain metabolically activity depend on blood oxygen level-dependent (BOLD) signal which in very different from PET technology. The diverse methods included could be a source of heterogeneity between studies in that we only include PET studies. Stuhrmanm et al [40] only described the changed brain region in their paper not using meta-analysis methods. Our meta-analysis focuses on glucose metabolism PET studies and included Chinese articles thus compiling a larger more homogeneous overall sample.

Although meta-analysis results across studies can help us to identify consistent findings in the literature, they may lack specificity as to the nature of any abnormality described. Insula, limbic system, basal ganglia, thalamus, and cerebellar activity changes are also found in other mental illness [15,41-43]. Several limitations need to be considered in the interpretation of our results. Firstly, methodological differences in PET recording and measurements may result in the heterogeneity among the different studies [1] and the limitation of ALE meta-analysis method [19]. However, ALE is widely accepted method used in neuroimaging meta-analysis which could correct for multiple comparisons and is different from traditional meta-analysis. And samples of three studies were geriatric depression and treatment-resistant depression patients. However, we only included FDG-PET studies which should lead to a more homogenous sample. Secondly, publication bias affecting the primary studies [44] could have influenced our results [45]. Last, it should be noted that our results may be limited by a small number of brain metabolism studies, the ALE technique is very dependent on the number of foci included in the analysis [46]. However, we found significant ALE clusters that were consistent with the path physiology of MDD.

## Conclusion

In conclusion, our meta-analysis involving brain metabolism PET studies in MDD patients showed dysfunctional regions including insula, limbic system, basal ganglia, thalamus, and cerebellum that has been previously proposed in depression. In particular, decreased insula activity might play a key role in the neuropathology of depression, which is compensated by increased metabolism in thalamus and cerebellum. However, these results lack specificity as they are similar to other neuropsychiatric diseases such as bipolar disorders and schizophrenia. Further research is required to find the specific brain regions which are the primary physiological changes and which are the secondary changes involved in the neuropsychiatric network of depression.
